# Prostate cancer - evidence of exercise and nutrition trial (PrEvENT): study protocol for a randomised controlled feasibility trial

**DOI:** 10.1186/s13063-016-1248-x

**Published:** 2016-03-07

**Authors:** Lucy Hackshaw-McGeagh, J. Athene Lane, Raj Persad, David Gillatt, Jeff M. P. Holly, Anthony Koupparis, Edward Rowe, Lyndsey Johnston, Jenny Cloete, Constance Shiridzinomwa, Paul Abrams, Chris M. Penfold, Amit Bahl, Jon Oxley, Claire M. Perks, Richard Martin

**Affiliations:** NIHR Biomedical Research Unit in Nutrition, Diet and Lifestyle, Level 3, University Hospitals Bristol Education Centre, Upper Maudlin Street, Bristol, BS2 8AE UK; School of Social and Community Medicine, University of Bristol, Canynge Hall, 39 Whatley Road, Bristol, BS8 2PS UK; Bristol Urological Institute, North Bristol NHS Trust, Southmead Hospital, Southmead Way, Westbury-on-trym, Bristol, Avon BS10 5NB UK; School of Clinical Sciences, University of Bristol, Southmead Hospital, Southmead Way, Westbury-on-trym, Bristol, BS10 5NB UK; North Bristol NHS Trust, Southmead Hospital, Southmead Way, Westbury-on-trym, Bristol, Avon BS10 5NB UK; University Hospital Bristol NHS Trust, Bristol Haematology and Oncology Centre, Horfield Road, Bristol, BS2 8ED UK; Cellular Pathology, North Bristol NHS Trust, Southmead Hospital, Southmead Way, Westbury-on-trym, Bristol, Avon BS10 5NB UK

**Keywords:** Cohort, Randomised control trial, Prostate cancer, Radical prostatectomy, Physical activity, Nutrition, Intervention

## Abstract

**Background:**

A growing body of observational evidence suggests that nutritional and physical activity interventions are associated with beneficial outcomes for men with prostate cancer, including brisk walking, lycopene intake, increased fruit and vegetable intake and reduced dairy consumption. However, randomised controlled trial data are limited. The ‘Prostate Cancer: Evidence of Exercise and Nutrition Trial’ investigates the feasibility of recruiting and randomising men diagnosed with localised prostate cancer and eligible for radical prostatectomy to interventions that modify nutrition and physical activity. The primary outcomes are randomisation rates and adherence to the interventions at 6 months following randomisation. The secondary outcomes are intervention tolerability, trial retention, change in prostate specific antigen level, change in diet, change in general physical activity levels, insulin-like growth factor levels, and a range of related outcomes, including quality of life measures.

**Methods/design:**

The trial is factorial, randomising men to both a physical activity (brisk walking or control) and nutritional (lycopene supplementation or increased fruit and vegetables with reduced dairy consumption or control) intervention. The trial has two phases: men are enrolled into a cohort study prior to radical prostatectomy, and then consented after radical prostatectomy into a randomised controlled trial. Data are collected at four time points (cohort baseline, true trial baseline and 3 and 6 months post-randomisation).

**Discussion:**

The Prostate Cancer: Evidence of Exercise and Nutrition Trial aims to determine whether men with localised prostate cancer who are scheduled for radical prostatectomy can be recruited into a cohort and subsequently randomised to a 6-month nutrition and physical activity intervention trial. If successful, this feasibility trial will inform a larger trial to investigate whether this population will gain clinical benefit from long-term nutritional and physical activity interventions post-surgery.

Prostate Cancer: Evidence of Exercise and Nutrition Trial (PrEvENT) is registered on the ISRCTN registry, ref number ISRCTN99048944. Date of registration 17 November 2014.

## Background

Prostate cancer is the most common malignancy in men in the Western world [1], accounting for a quarter of all new male cancer cases in the United Kingdom [2]. In many cases, localised prostate cancer is slow growing; however, an unpredictable proportion of men suffer more rapidly progressive, fatal disease.

A growing body of observational evidence suggests that certain nutritional and physical activity interventions are associated with beneficial outcomes for men with prostate cancer, such as improved quality of life, longer disease-free survival and reduced prostate cancer mortality [3, 4]. In addition, a cancer diagnosis can provide a teachable moment during which an individual may be motivated to improve their health behaviours [5, 6].

The Prostate Cancer: Evidence of Exercise and Nutrition Trial (PrEvENT) is a feasibility randomised controlled trial (RCT). PrEvENT investigates whether men who have undergone radical prostatectomy will adhere to a nutritional and physical activity intervention and explores the implications of the intervention on a range of surrogate outcomes.

The rationale for the interventions included in the trial is described below.

### Physical activity

Observational studies suggest that moderate to vigorous physical activity is associated with reduced risk of biochemical recurrence and mortality in men with prostate cancer. For example 3 hours of moderate to vigorous physical activity per week was associated with a 61 % decrease in prostate cancer mortality compared with less than 1 hour [7]. Prostate cancer most often becomes evident in older age when men may not always be able, or motivated, to participate in vigorous activity. Brisk walking is a potential alternative, as it provides exposure to moderate-intensity activity [8], and has been associated with anti-cancer cellular behaviour [9]. Brisk walking does not require specialised equipment or training and is not location specific. A higher body mass index has been associated with poorer prostate cancer survival suggesting that promotion of physical activity may reduce cancer progression through weight control [10]. Guidelines recommend that people with cancer do 30 minutes of moderate-intensity exercise 5 days per week [11]. However, the majority of men with prostate cancer do not meet these guidelines [12].

### Lycopene

Lycopene is a carotenoid constituent of tomatoes with potential anti-cancer activity. In an observational study, men’s intake of tomato sauce following a diagnosis of prostate cancer was associated with reduced risk of disease progression [13]. A small trial (n = 81) in men at high risk of prostate cancer demonstrated the acceptability and tolerability of lycopene supplements as tablets [14]. However, there is limited evidence from RCTs for the effect of lycopene when used as adjunct therapy in men with prostate cancer [15].

### Plant-based diets

Vegetables and legumes have been associated with a lower risk of prostate cancer, and in some studies, they are associated with a reduced risk of advanced or aggressive disease at diagnosis [10, 16]. They are thought to enhance immunity and inhibit cell growth, and consumption after diagnosis is associated with a reduced risk of progression [17, 18]. Evidence of the relationship between fruit intake and prostate cancer progression is limited. However, both fruit and cruciferous vegetables are high in vitamin C and increased intake of vitamin C has been associated with clinical outcomes, such as reduced prostate cancer risk [19], DNA repair [20] and maintained levels of PSA in men with biochemical recurrence of prostate cancer [21].

### Dairy products and soy milk

Observational evidence suggests that high intakes of dairy products and calcium, for example from cow’s milk, could be associated with a greater risk of prostate cancer [22, 23], but the evidence is inconclusive [10, 24]. Prostate cancer has been inversely associated with intake of soya and soya foods [25, 26]. Studies have reported decreased mean (PSA) levels in men with prostate cancer, who were supplemented with soy bread or soy and linseed bread, compared to supplementation with wheat bread [27]. Not all trials, however, have reported such PSA reductions in association with soy protein supplementation [28].

### Empirical data informing the trial protocol

We conducted interviews to explore attitudes towards, and previous experience of, nutrition and physical activity interventions, as well as opinions about the proposed PrEvENT trial, with men following radical prostatectomy for prostate cancer, their partners and their healthcare professionals. These interviews provided evidence that although some men may not have much experience of such interventions, they would attempt to make changes, especially if they felt it would benefit them or influence future prostate cancer treatment. They were positive about the proposed intervention. However, potential barriers that the men highlighted, including treatment-related incontinence and aversions to certain food products were discussed and considered when developing the PrEvENT intervention. Results from the qualitative interviews are to be published elsewhere (Unpublished observations, Hackshaw-McGeagh et al.).

As well as incorporating the World Cancer Research Fund Continuous Update Project review [10], we undertook a systematic review of the existing literature on the effect of dietary, nutritional and physical activity interventions on clinical outcomes in men with prostate cancer [29]. This review highlights the paucity of good quality RCT evidence and supports the development of feasibility and pilot RCTs to underpin the development of definitive trials in this area.

Based on existing evidence, we hypothesised that following radical prostatectomy for localised prostate cancer, men will accept, and adhere to, nutrition and physical activity interventions. The feasibility trial was developed with the intention of informing a larger trial of these interventions.

## Methods/design

### Trial setting and participants

The PrEvENT trial has a 2 x 3 factorial design (resulting in six different experimental conditions), where men are randomised to levels of both a physical activity and nutritional intervention. Randomisation is carried out by the Bristol Randomised Trial Collaboration [30] using a web based system. Participants and the research team are not blinded; only the trial statistician will be blinded. The trial is being conducted under the auspices of the National Institute for Health Research (NIHR) Bristol Biomedical Research Unit in Nutrition, Diet and Lifestyle [31]. The feasibility trial is being conducted at the North Bristol National Health Service (NHS) Trust. Inclusion and exclusion criteria are listed in Table 1.

**Table 1:** PrEvENT inclusion and exclusion criteria

Inclusion criteria
• Clinically localised prostate cancer
• Listed for radical prostatectomy
• Capacity to provide informed consent
• Aged 18 or over
• Sufficient understanding of the English language
Exclusion criteria
• Unable to provide informed consent
• Unsuitable to participate following guidance from their clinician
• Unable to undergo follow-up
• Factors that constrain participation in any aspect of the intervention, including:
o Co-morbidities, for example:
▪ Congestive heart failure or angina, recent myocardial infarction or breathing difficulties requiring oxygen use or hospitalisation
o Unable to walk without the aid of a mobility aid (other than a walking stick)
o Religious beliefs
o Allergy to lycopene
• Current heavy consumers of the nutritional element of the intervention, that is, taking daily lycopene supplements for more than 3 months
• Current participation in high levels of regular physical activity, for example doing strenuous activity five or more times a week for long enough to work up a sweat
• Any additional reason for not being able to participate in any aspect of the intervention

### Trial design

**Fig. 1:**
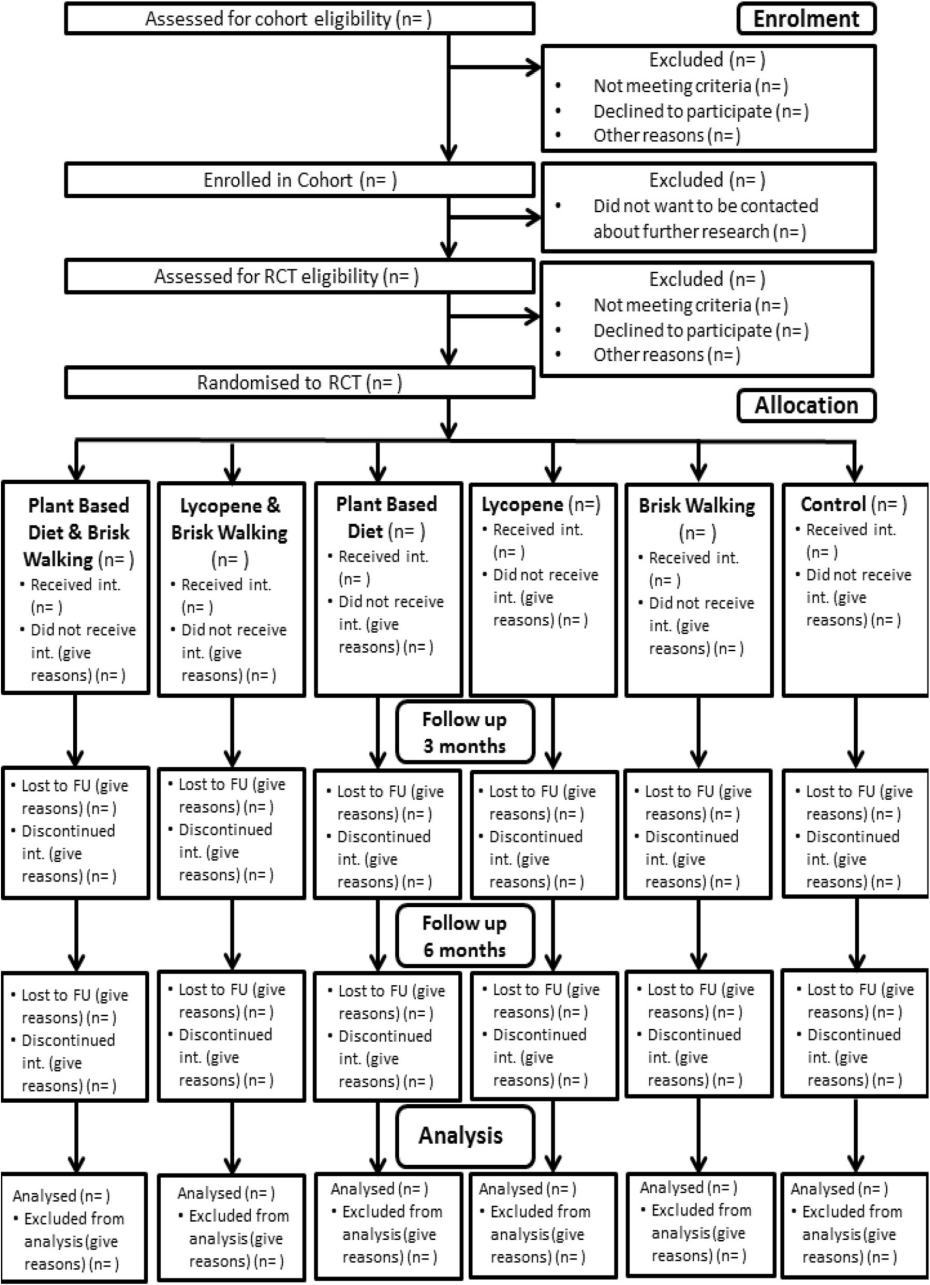
PrEvENT CONSORT flow diagram

This trial has two phases: i) recruitment of men scheduled for radical prostatectomy into a cohort for baseline and tissue markers and ii) post-radical prostatectomy recruitment into an RCT. The CONSORT flow diagram, illustrating passage through the RCT, is shown in Fig. 1.

### Initial recruitment phase

Men are recruited to the trial via a consecutive opportunistic sample; that is, men who are conveniently accessible to the research team in the urology outpatients clinic and who meet the inclusion criteria are invited to participate until the sample size is reached and then are introduced to the study by a member of the clinical care team at the appointment where a decision about treatment is made. The expected duration between the appointment where a decision about treatment is made and surgery is approximately 4 weeks. Men who indicate an interest in the trial are directed to the research nurse for further information and given an initial research appointment where informed consent into the cohort is obtained and key data collected. Data that are obtained include i) a baseline questionnaire, including collection of information about diet, physical activity levels, mood and prostate symptoms and ii) blood samples used to assess baseline levels of nutritional biomarkers and biomarkers of prostate cancer progression, such as PSA. Men who consent will allow prostate tissue samples to be obtained from the prostate that is removed at surgery. The tissue is stored for future analysis. Full details of data collected at each time point can be found in Table 2. Each man’s National Health Service (NHS) number is also recorded for anonymous data linkage to routine NHS electronic datasets, such as the Cancer Registries and the Office for National Statistics Vital Statistics dataset. Recruitment into the cohort study will continue until the specified sample size (n = 100) has been reached.

**Table 2:** Data collected during PrEvENT

Participant assessment	Who					When							
	Usual care team	Consultant	Research nurse	Participant	Research team	Pre-screening	Cohort baseline appointment	Surgery	Cohort follow-up/true trial baseline appointment	6-month intervention duration	3-month follow-up appointment	6-month follow-up appointment	Evaluation interview
Eligibility screening	X	X	X			X							
Informed consent			X	X			X		X				
Demographic data				X			X		X		X	X	
Family medical history				X			X		X		X	X	
Anthropometric data weight, height, % body fat			X				X		X		X	X	
Blood sample			X				X		X			X	
Diet data FFQ [43]				X			X		X		X	X	
Physical activity data Recent Physical Activity Questionnaire [44]				X			X		X		X	X	
Urinary symptoms ICSmale-SF [45]				X			X		X		X	X	
Psychological measures POMS-SF [46] and Benefit Finding Scale [47]				X			X		X		X	X	
Health beliefs data Adapted from [37] and [32]				X			X		X		X	X	
Quality of life measures FACT-P [48]				X			X		X		X	X	
General health data SF-12 [49]				X			X		X		X	X	
Cancer related fatigue FACIT-Fatigue [48]				X			X		X		X	X	
Lifestyle data Drinking and smoking				X			X		X		X	X	
Accelerometry data				X			X		X			X	
Prostate tissue		X						X					
Pedometer data				X						X			
Daily monitoring data Step count, record of fruit, vegetable, dairy and lycopene intake				X						X			
Qualitative evaluation data				X	X								X

At 5 weeks post-surgery, all men are posted a follow-up data collection questionnaire and invited to a second appointment with the research nurse.

### Recruitment into the randomised control trial

Men invited to participate in the RCT will have agreed to be contacted about further related research during the initial cohort recruitment phase. Along with the post-surgery follow-up data collection questionnaire, these men receive an invitation to participate in the RCT. They are telephoned by the research nurse and screened for trial eligibility. Inclusion and exclusion criteria for the RCT can be seen in Table 1. Where eligible, a research appointment is arranged for the following week, where a consent form is completed along with collection of blood samples and provision of an accelerometer (to capture intensity of physical activity) to wear continuously for the following week. All men are also provided with a pedometer (to measure daily step count), to wear for the 6-month duration of the trial. The data items that are being collected are listed in Table 2.

The men are randomised into both the nutritional and physical activity intervention arms of the trial and provided with instructions specific to their intervention allocation. They receive texts, emails and post relating to the trial interventions, including recipe ideas and motivational messages, as applicable to their allocated intervention, to encourage continuation of their trial arm intervention throughout the 6-month trial period. These contacts take place at 1, 2, 5, 8, 13, 15 and 18 weeks post-randomisation. They include gratitude for ongoing support of the research and a reminder of the importance of the data collected. Men are reminded of the changes they have been asked to make and the data they have been requested to record daily. Instructions are given of how to carry out the behaviour change, as well as being reminded of the potential benefits of the new behaviour, and men are informed that others similar to them carry out the intended behaviour. Message content was developed in line with the Theory of Planned Behaviour [32] (See Theoretical Underpinning below).

At 3 and 6 months following randomisation, the men attend a follow-up research appointment, where repeat questionnaires are completed. At the 6-month follow-up appointment, a blood sample is taken. For the final week of the trial, men wear an accelerometer to record the intensity of their physical activity.

We intend to recruit 100 men to the cohort, and of these, randomise at least 80 to the randomised controlled trial.

### Evaluative interviews

Six months following randomisation, a random sample of men are interviewed to explore their experiences of the changes they were asked to make to their nutrition and physical activity, and their experience of participating in the research.

### Withdrawal and discontinuation of participants

Men may be withdrawn from the study by their clinical care team or the research team if it is considered detrimental to continue, for example in light of disease progression. Men who fail to attend appointments are contacted via telephone to encourage them to attend, to arrange alternative appointments and to determine reasons for withdrawal.

### Interventions

In the nutritional intervention, men are randomised to either a lycopene supplement intervention or a plant-based intervention or a nutritional control arm. The lycopene intervention involves one 10 mg lycopene capsule daily (provided to the man). The plant-based intervention comprises eating as many portions of fruit and vegetables daily as possible, aiming for at least five portions, and swapping dairy milk for non-dairy alternatives, for example, soya milk, as often as the men can. The control arm men carry on with their usual diet; if the men ask for nutritional advice, standard, publically available, information is provided.

In the physical activity intervention, men are randomised to either walking at a brisk pace for 30 minutes, on at least five days a week, on top of their usual physical activity or to continue with their usual levels of physical activity.

The men are provided with materials and detailed instructions specific to their allocated intervention arm by trained research nurses and asked to follow their allocated intervention arm for 6 months.

### Primary outcome measures

Co-primary outcomes are randomisation rates (the proportion of eligible men who agree to be randomised) where a rate of 65 % or above would be considered acceptable [33] and adherence to the interventions at 6 months following randomisation, where a rate of 75 % or above would be considered acceptable. Adherence will be calculated for both the nutrition and physical activity interventions and is taken to be following intervention instructions > 90 % of the time. Adherence to the nutrition intervention will be assessed by participant self-report on the daily monitoring form. This form is completed daily by the participants for the duration of the 6-month intervention. For the lycopene arm, men are asked if they took their supplement daily - yes/no; for vegetable and fruit consumption, men are asked how many portions they consumed daily; for dairy milk consumption, men are asked how much of their dairy milk they replaced with an alternative – all/some/none. Adherence to the physical activity intervention will be assessed by their self-reported adherence to the recommended minimum 30 minutes of brisk walking five times per week during the 6-month intervention phase.

In order to assess the adherence rate with a confidence interval of ± 5 % and an estimated expected adherence rate of 75 %, the required minimum sample size for this feasibility study would be approximately 75 participants [34]. Incorporating an expected randomisation rate of 80 %, we intend to recruit at least 100 men into this study.

### Secondary outcome measures

The secondary outcomes include intervention tolerability (based on qualitative interviews and reported adverse events), retention (men successfully followed up at 6 months, as a proportion of those who were recruited to the trial and randomised into a study arm), change in PSA (a protein produced by cells of the prostate gland, which is often elevated in men with prostate cancer) [35] and insulin-like growth factor (IGF-I) levels (raised IGF-I levels are increasingly being implicated as a potential risk factor for cancer) [36] and change in all other data collected between intervention groups at the different time points. We will explore whether biomarkers of lycopene for the lycopene intervention arm, alpha-carotene, beta-carotene, lutein, lycopene, total carotenoids and vitamin C for fruit and vegetable intake, and phyto-oestrogen, as a biomarker for soya milk, are affected by the proposed interventions. Full details of the data being collected at specific time points are described in Table 2. The study was not powered for secondary outcomes.

### Tissue collection

All samples are collected, used and stored in accordance with the Human Tissue Act 2004. Prostate tissue sections are formalin-fixed and tissue that is surplus to diagnostic requirements is stored for later analysis of nutritional and physical activity biomarkers (including epigenetic and metabolomic biomarkers) and surrogate biomarkers of prostate cancer (for example, expression levels of IGF-I). Stage, margin status and Gleason score for each radical prostatectomy specimen will be recorded. Collection of blood samples will allow biological mechanisms linking nutritional, physical activity and other lifestyle factors with prostate cancer risk and progression to be further investigated.

### Theoretical underpinning

Three psychological theories will be employed during the PrEvENT trial, each serving a unique purpose. These are ‘Teachable Moments’ [5], the Transtheoretical Model of Change (Stages of Change) [37] and the Theory of Planned Behaviour [32].

Data analysis will allow exploration of whether men with prostate cancer make naturally occurring changes to their diet and physical activity as a result of their cancer diagnosis and treatment. This will assist in establishing whether receiving a diagnosis of prostate cancer and undergoing treatment can be utilised as a ‘Teachable Moment’ [5]. It is hypothesised that prostate cancer diagnosis is a health event, or Teachable Moment, thought to motivate individuals to make changes to health or lifestyle behaviour.

The Transtheoretical Model of Change (Stages of Change) [37], assesses an individual’s level of readiness to change. The model categorises people into a number of stages. Men in the trial will have their readiness to change assessed at the four data collection time points. Analysis will then establish whether men, at different levels of readiness to change, respond differently to the intervention. Additionally, it will be established whether the men’s level of readiness to change alters during the trial.

The Theory of Planned Behaviour [32] explores three key considerations which are proposed to influence whether an individual will make a behaviour change or not. These are behavioural beliefs, normative beliefs and control beliefs, which combine to influence intention to change behaviour. The PrEvENT trial participant-facing documents have been developed in a manner that intends to influence these three theoretical considerations, thus helping to implement the behaviour change in the men, and improve adherence and acceptability of the interventions.

### Analysis plan

The primary comparative analyses will be by intention-to-treat. Baseline characteristics of each group will be tabulated using means and standard deviations for normally distributed data, medians and interquartile ranges for non-normally distributed data, and percentages and counts for categorical data. Randomisation rate and adherence to the intervention will both be reported as a percentage, along with the respective 95 % confidence interval.

We will also conduct exploratory analyses of change in physical activity and fruit and vegetable consumption (as measured for adherence to the interventions) will be analysed using a two-way ANOVAs or appropriate non-parametric tests if necessary. Change in selected secondary outcomes (IGF-I, PSA and blood biomarkers) will be analysed similarly, although this study is not powered for secondary outcomes. These analyses will focus on the main effects of the interventions, but we will also explore possible interactions between the interventions with respect to these outcomes. Assuming 80 men are recruited into the trial, this will provide approximately 90 % power to detect a large main effect size (f = 0.4) with an alpha level of 0.05, analysing the study as a 2 x 3 factorial design (power will be reduced after correcting for multiple testing). We will also explore associations between baseline Stage of Change; behavioural, normative and control beliefs; and adherence to the interventions using chi-square tests and logistic regression models.

Qualitative data collected during this research will be analysed thematically using a qualitative method, such as the framework approach [38]. Individuals will not be identified by name in the written transcript. A qualitative analysis computer programme, such as NVivo [39], will be used to assist analysis.

The SPIRIT 2013 checklist [40] and the CONSORT statement [41] were followed and adhered to during the development of the trial protocol and implementation. The PrEvENT trial has been given full ethical approval by Cornwall and Plymouth Research Ethics Committee (REC), ref: 14/SW/0056, and registered on the ISRCTN registry, ref number ISRCTN99048944. All participants provide fully informed consent.

## Discussion

**Fig. 2:**
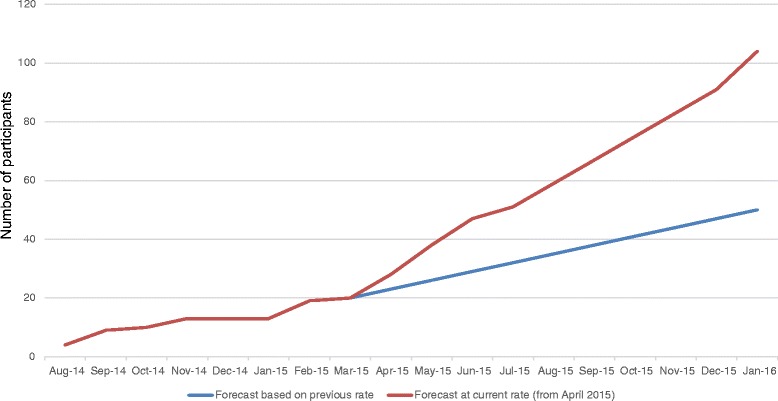
PrEvENT recruitment forecast

Recruitment began in August 2014 (14 months previous to the time of manuscript submission), and to date, 69 men have been recruited. Initially, recruitment was intended to be complete within a 6-month period. However, this is now acknowledged to be an ambitious estimate. The initial recruitment approach, as described previously, was at the appointment where a decision about treatment is made, where eligible men would be invited into the research by the clinical staff during this appointment. Initial rates of recruitment were, however, slower than anticipated, with three men being recruited on average per month, primarily due to overburden of the clinical staff. Thus, a different approach to recruitment is now being taken. Eligible men are identified through a process of theatre list screening by the research nurse. Eligible men are contacted via a consultant-endorsed letter and then telephoned by the research nurse to be invited into the research. This method is proving to be fruitful, and recruitment rates have increased to eight men recruited on average per month. We now anticipate recruitment to be complete by January 2016. This is illustrated in Fig. 2.

### Summary

The PrEvENT RCT aims to determine whether men with localised prostate cancer, who are scheduled to undergo radical prostatectomy, can be recruited into a pre-surgery cohort and subsequently randomised to a 6-month post-surgery diet and physical activity intervention trial. The research additionally aims to determine whether this population will adhere to their intervention arm, complete trial documentation as requested and be retained in the research until the conclusion of the trial. Conclusions from this feasibility trial, along with previous literature relating to nutrition and physical activity associations with prostate cancer risk [29, 42], are intended to inform a larger longitudinal trial to investigate whether men with localised prostate cancer, who have undergone radical prostatectomy, will benefit clinically from long-term diet and physical activity interventions.

### Trial status

The trial began recruitment in August 2014 and recruitment was ongoing at the time of submission.

## Abbreviations

FACIT: Fatigue Functional Assessment of Chronic Illness Therapy-Fatigue
FACT-P: Functional Assessment of Cancer Therapy-Prostate
FFQ: Food frequency questionnaire
FU: follow-up
ICSmale-SF: International Continence Society male-Short Form
IGF: insulin-like growth factor
Int.: intervention
n: Number
NHS: National Health Service
NIHR: National Institute for Health Research
POMS: Profile of Mood States – Short Form
PrEvENT: Prostate cancer: Evidence of Nutrition and Exercise Trial
PSA: prostate-specific antigen
RCT: randomised control trial
SF12: Short Form Health Survey
